# Cardiovascular risk factors of airport visitors in India: results from a nation‐wide campaign

**DOI:** 10.1111/jch.14413

**Published:** 2021-12-13

**Authors:** Willem J. Verberk, Nilesh Goswami

**Affiliations:** ^1^ CARIM School for Cardiovascular Diseases Maastricht University Maastricht the Netherlands; ^2^ Eris Lifesciences Ltd. Ahmedabad India

**Keywords:** cardiovascular risk, diabetes, hypertension, obesity, screening

## Abstract

Cardiovascular diseases have become the major cause of death in India, but overall awareness is still low. Therefore, the initiative was undertaken to set up health care screening booths at eight airports and one hospital throughout India to increase awareness and to determine cardiovascular risk factors. Participants were screened for hypertension (systolic blood pressure (BP) ≥140 mmHg or diastolic BP≥90 mmHg), diabetes [fasting blood glucose (FBG) level ≥126  or ≥200 mg/dL for random blood glucose (RBG)], and body mass index (BMI). Among 100 107 participants screened (46 ± 13 years; 17% women), prevalence of diabetes was 12 571 (15%), hypertension: 30 345 (33%) and overweight: 61 219 (65%). Diabetes was treated more often than hypertension (44% vs 11%). Hypertension and diabetes prevalence values were relatively high in young obese adults; BMI correlated significantly (*p <* .001) stronger to both systolic BP and RBG for subjects younger than 40 years than for those who were older (*r* = 0.27 vs *r* = 0.06 and *r* = 0.15 vs *r* = 0.03, respectively). Among obese women aged 60 years and older the hypertension prevalence was higher than 40%, in obese men this prevalence value was already seen from the group of 30 to 40 years old. For participants older than 50 years with hypertension, diabetes prevalence was 20%. These results show that screening initiatives like these are highly needed to increase the overall awareness of diabetes and particularly of hypertension. Systematic screening programs also help to identify specific patient populations and cope with undertreatment of those at the highest cardiovascular risk. The fact that women were underrepresented in the present screening campaign suggests actions are needed to encourage them to participate in health care programs.

## 1 INTRODUCTION

India is a rapidly developing country; this has led to a rapid change from reduction in communicable diseases to an increase in non‐communicable diseases. Following this, cardiovascular diseases have become the number one cause of death, and responsible for a quarter of all mortality.^1^ This number is still increasing due to factors related to developing such as industrialization, urbanization, and changes in lifestyle, so‐called epidemiological transition.^2^ A nationally representative study among 1.3 million Indians showed prevalence values of diabetes and hypertension of 7.5% and 25%, respectively.^3^ In addition, the prevalence of people with diabetes in India had increased from 26 million in 1990 to 65 million in 2016^4^ with a prevalence of 12% in those older than 50 years of age.^5^ Consequently, cardiovascular diseases are responsible for a massive economic burden with estimated costs of $2.17 trillion between 2012 and 2030 being the major cause of the economic loss.^6^ However, despite these high figures, awareness of both diabetes^7^ and hypertension^8^ is still low among Indians.

Public screening activities may help to increase awareness of cardiovascular diseases that are often symptomless and may improve the insight into the prevalence of these diseases and its control. Following this, an initiative was undertaken to set up health care screening booths in eight airports and one hospital around India. The current paper presents the obtained data of more than 100 000 participants screened for cardiovascular risks.

## 2 METHODS

### 2.1 Study design and participants

The study design envisaged a retrospective analysis of a database created from a community‐based screening campaign focusing on blood pressure (BP), blood glucose measurement and body mass index (BMI), and the collection of basic information.

Data were obtained in the period between August 2019 and March 2020. A visible temporary booth was established at 8 airports (Ahmedabad, Cochin, Coimbatore, Delhi Metro, Goa, Lucknow, Mumbai, and Trivandrum) and one hospital (KG Hospital Coimbatore).

Approval for this retrospective study was obtained from Ripon Independent Ethics Committee (RIEC), Chennai, India. A digital signature was obtained from each participant while data were electronically collected using a tablet. Data from the participants were collected in a non‐identifiable manner to protect the privacy of all participants. The number of participants screened per state of India can be found in Figure Supplement S1.

### 2.2 Participants

**FIGURE 1:**
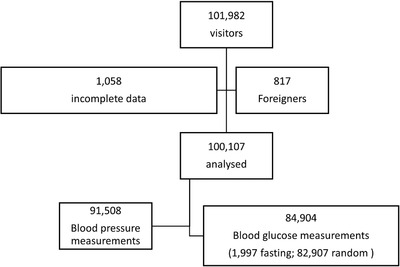
Flow chart of the study

The screened population consisted of an unselected sample of 101,982 participants. Since most of the screening was done at the airport, the visitors could come from anywhere. However, it appeared that only 0.8% (817) of all visitors were non‐Indians. As the present analysis was aimed at improving the insight into the prevalence of cardiovascular diseases in India, non‐Indians were excluded from the analysis for the present study. A table in which the characteristics of subjects from “elsewhere” were compared to those with residence in India are shown in the Table S1. In total, 100 107 Indian participants were analyzed for the present study (see Figure 1, flow chart).

### 2.3 Booth visit

When entering the booth to be measured the following participant's characteristics were registered: sex, age, nationality, city of residence, profession, and flying frequency. In addition, participants were asked if hypertension or diabetes was diagnosed previously and in all except three booths (Cochin, Coimbatore, Delhi Metro), it was registered if participants received treatment for either one of these conditions.

### 2.4 Measurements

#### 2.4.1 Blood pressure measurement

The booth attendants were well trained to take BP and blood glucose readings as per routinely followed protocols at clinic or lab. BP was measured with a validated oscillometric BP monitor that automatically measured 3 times in a row with 15‐s interval times and provided the average of three BP readings (Circa 120/80, Watch BP Home A, Eris Ahmedabad).^9^, ^10^, ^11^ Patients were measured in a sitting position, with their back supported and the arm supported at heart level. Participants were instructed not to talk during BP measurement and keep their legs uncrossed with both feet flat on the floor. A cuff‐size was selected appropriate to the participant's arm circumference (small size cuffs for upper arm circumferences between 14 and 22 cm, medium size 22‐ 32 cm, large size 32–42 cm, and extra‐large size 32–52 cm).

#### 2.4.2 Blood glucose measurement

After the BP measurement had been taken, blood glucose was determined using the Accu‐Chek Performa blood glucose monitor (Roche, Germany). Participants were asked when they had last eaten. If the participant reported not to have eaten within the last 8 h their glucose value was considered as Fasting Blood Glucose (FBG), all other values were considered Random Blood Glucose (RBG).

Thereafter, participants were asked to take off their shoes for measuring their height (Thermocare, height measurement scale) and weight (Omron weighing scale) to assess BMI.

Results were discussed with the participant and appropriate advice followed the obtained measurements (for example, participants were recommended to visit their doctor if the values obtained fell outside the normality range). Finally, a printed report was handed to the participants or sent by SMS if this was preferred (see Figure S2).

### 2.5 Statistical analysis

Participants were classified as hypertensive if the average of three BP readings was equal to or higher than 140 mmHg and /or 90 mmHg for systolic and diastolic BP.^12^ Subsequently, BP values were categorized according to ESH/ESC ‐standards: (Optimal < 120 and < 80 mmHg, Normal 120–129 and 80–84 mmHg, High normal 130–139 mmHg and 85–89 mmHg Grade 1 hypertension: 140–159 and 90–99 mmHg, Grade 2 hypertension 160–179 and 100–109 mmHg, Grade 3 hypertension: ≥180 and ≥110 mmHg for systolic and diastolic blood pressure, respectively).^12^

For classifications in glucose ranges guidelines of the American Diabetes Association were applied. For FBG a level of ≥ 126 mg/dL and for RBG a value ≥ 200 mg/dL was labeled as diabetes.^13^ Weight was divided in 6 classes (underweight, BMI less than 18.5 kg/m^2^; normal, 18.5‐24.9 kg/m^2^; overweight, 25.0 to 29.9 kg/m^2^; class I obesity 30–34.9 kg/m^2^, class II obesity 35–39.9 kg/m^2^, class III obesity 40 kg/m^2^ or higher). Prevalence for overweight (yes, no [≥ 25.0 kg/m^2^]) and obesity (yes, no [≥ 30.0 kg/m^2^]) were determined and used for comparison. Participants were divided in age groups of less than 30 years old with stepwise 10‐years increases until 60 years and older.

Participant's characteristics were compared for sex and other aspects such as screening location (hospital vs airports), BP and weight class. Main demographic and clinical data were summarized by calculating the mean (±SD) in case of continuous variables and the absolute (n) and relative (%) frequency in case of categorical variables. Differences across groups were evaluated using retrospective analysis of variance (ANOVA). For categorical variables, differences across groups were evaluated using Chi‐square tests. We calculated the prevalence of hypertension and diabetes for categories of age, BP (for diabetes prevalence), and BMI. To determine the statistical relationship between systolic BP and age and other measured parameters, Pearson's correlation coefficients (CCs) and confidence intervals were calculated. In order to verify if Pearson's CCs would differ between groups younger and older than 40 years of age, CCs were calculated for systolic BP and BMI and RBG and BMI and compared for significant differences. No methods for imputation were performed. Results were presented in *p*‐values if considered relevant, a *p* value of < .01 was considered significant. Analyses were performed using the statistical package R Studio Version 1.2.5033 for Windows.

## 3 RESULTS

Data from 100 107 Indian visitors were collected with an average age of 45.9 ± 12.9 years. Among these, there were 8599 (8.6%) who did not undergo BP measurement and 15 203 (15.2%) participants had no blood glucose measurement.

Table 1 shows the unweighted characteristics of all booth visitors and separated for sex (17% female). In total, 30 345 (33.2%) had hypertension and 12 571 participants (14.8%) were classified as diabetes (388 [19.4%] based on FPG and 12 183 [14.7%] based on RBG). Weight and height measurements showed an average BMI of 26.7 ± 4.0 kg/m^2^, 61 219 (64.8%) participants were overweight, and 17 074 (18.1%) were obese.

**TABLE 1:** Subject characteristics separated for sex

	Female (No. = 17 321)	Male (No. = 82 786)	Total (No. = 100 107)	*p*
Age [y]				< .001
	45.3 (13.8)	46.0 (12.6)	45.9 (12.9)	
Systolic BP [mmHg]				< .001
	123.4 (18.7)	131.0 (16.6)	129.7 (17.2)	
Diastolic BP [mmHg]				< .001
	76.7 (10.7)	83.6 (10.6)	82.4 (11.0)	
Heart Rate [BPM]				< .001
	84.9 (12.8)	85.5 (13.6)	85.4 (13.5)	
Random Blood Glucose [mg/dL]				< .001
(no. = 82 907)	139.1 (58.6)	147.9 (65.1)	146.5 (64.2)	
Fasting Plasma Glucose [mg/dL]				.053
(no. = 1997)	108.0 (23.8)	110.4 (24.6)	110.0 (24.5)	
Height [cm]				< .001
	158.9 (6.4)	170.7 (7.2)	168.6 (8.3)	
Weight [kg]				< .001
	66.5 (12.0)	78.0 (12.5)	75.9 (13.2)	
Body Mass Index [kg/m^2^]				< .001
	26.3 (4.5)	26.7 (3.9)	26.7 (4.0)	
Overweight				< .001
Non‐Overweight	6688 (40.6%)	26 548 (34.0%)	33 236 (35.2%)	
Overweight	9780 (59.4%)	51 439 (66.0%)	61 219 (64.8%)	
Obesity				< .001
Non‐Obese	13 261 (80.5%)	64 120 (82.2%)	77 381 (81.9%)	
Obese	3207 (19.5%)	13 867 (17.8%)	17 074 (18.1%)	
Random Blood Glucose				< .001
Diabetes	1463 (10.8%)	10 720 (15.5%)	12 183 (14.7%)	
Non‐Diabetes	12138 (89.2%)	58 586 (84.5%)	70724 (85.3%)	
Fasting Plasma Glucose				.088
Diabetes	54 (16.1%)	334 (20.1%)	388 (19.4%)	
Non‐Diabetes	282 (83.9%)	1327 (79.9%)	1609 (80.6%)	
Blood pressure diagnosis				< .001
Hypertension	3470 (21.9%)	26 875 (35.5%)	30 345 (33.2%)	
Normotension	12 399 (78.1%)	48 765 (64.5%)	61 164 (66.8%)	
Diabetes total				< .001
DM	1517 (10.9%)	11 054 (15.6%)	12 571 (14.8%)	
non‐DM	12 420 (89.1%)	59 913 (84.4%)	72 333 (85.2%)	
Tachycardia [≥100 BPM]				< .001
Normal	13 829 (87.5%)	64 014 (85.0%)	77 843 (85.4%)	
Tachycardia	1967 (12.5%)	11 338 (15.0%)	13 305 (14.6%)	
Hypertension awareness				< .001
No	16 405 (94.7%)	77 748 (93.9%)	94 153 (94.1%)	
Yes	916 (5.3%)	5038 (6.1%)	5954 (5.9%)	
Diabetes awareness				< .001
No	16 360 (94.5%)	75 727 (91.5%)	92 087 (92.0%)	
Yes	961 (5.5%)	7059 (8.5%)	8020 (8.0%)	
Receiving treatment				< .001
Hypertension	568 (3.3%)	2840 (3.4%)	3408 (3.4%)	
Diabetes	600 (3.5%)	4865 (5.9%)	5465 (5.5%)	
Both	361 (2.1%)	2252 (2.7%)	2613 (2.6%)	
None	15 792 (91.2%)	72 829 (88.0%)	88 621 (88.5%)	
Profession				< .001
Business person	662 (4.5%)	19 444 (26.5%)	20 106 (22.8%)	
Corporate Job	5354 (36.1%)	45 648 (62.3%)	51 002 (57.9%)	
Education	214 (1.4%)	819 (1.1%)	1033 (1.2%)	
Government Service	611 (4.1%)	5278 (7.2%)	5889 (6.7%)	
Home Maker	7536 (50.8%)	1233 (1.7%)	8769 (10.0%)	
Student	458 (3.1%)	850 (1.2%)	1308 (1.5%)	
Flying Frequency				< .001
Occasional	13 221 (92.6%)	55 731 (77.2%)	68 952 (79.7%)	
Once a month	813 (5.7%)	12 000 (16.6%)	12 813 (14.8%)	
Once a week	146 (1.0%)	2692 (3.7%)	2838 (3.3%)	
Multiple times a week	104 (0.7%)	1805 (2.5%)	1909 (2.2%)	

Males were slightly older than females (46.0 ± 12.6 vs 45.3 ±13.8 years), had higher BMI (26.7 ±3.9 vs 26.3 ±4.5 kg/m^2^), higher RBG (147.9± 65.1 vs 139.1 ± 58.6 mg/dL), and higher systolic and diastolic BP values (131.0/ 83.6  vs 123.4/76.6 mmHg, all *p *< .001). In contradiction to the higher BMI for males, females showed higher obesity prevalence than males (19.5% vs 17.8% *p *< .001).

Of all participants 1,428 individuals were measured at a booth located at a hospital entrance hall, their characteristics slightly differed from the airport booth visitors (Table S2).

Table 2 shows that systolic BP had highest correlation values with age (*r* = 0.27), glucose (*r* = 0.17), and BMI (*r* = 0.16). FBG showed a stronger correlation with age (*r* = 0.29) and BMI (*r* = 0.16) than RBG (*r* = 0.22 and *r* = 0.09, respectively)

**TABLE 2:** Means, standard deviations, and correlations with confidence intervals for the parameters measured

Variable	*M*	*SD*	1	2	3	4	5	6
1. Age (years)	45.87	12.90						
2. BMI (kg/m^2^)	26.66	4.01	0.11					
			[0.11, 0.12]					
3. Systole (mmHg)	129.65	17.22	0.27	0.16				
			[0.26, 0.28]	[0.15, 0.16]				
4. Diastole (mmHg)	82.36	10.97	0.04	0.17	0.69			
			[0.03, 0.04]	[0.16, 0.17]	[0.69, 0.70]			
5. Heart rate (bpm)	85.39	13.47	‐0.08	0.12	0.04	0.22		
			[‐0.09, ‐0.08]	[0.11, 0.12]	[0.03, 0.05]	[0.22, 0.23]		
6. RBG (mg/dL)	146.37	64.08	0.22	0.09	0.17	0.07	0.22	
			[0.22, 0.23]	[0.08, 0.09]	[0.17, 0.18]	[0.06, 0.08]	[0.21, 0.23]	
7. FBG (mg/dL)	109.96	24.47	0.29	0.16	0.17	0.14	0.16	*NA*
			[0.25, 0.33]	[0.11, 0.20]	[0.12, 0.21]	[0.09, 0.18]	[0.11, 0.21]	

*Note. M* indicates mean; SD, standard deviation; FBG, fasting blood glucose; RBG, random blood glucose; NA, not applicable. Values in square brackets indicate the 95% confidence interval for each correlation. All correlation values are significant at *p* < .01.

### 3.1 Treatment

Among participants whose measured blood glucose value suggested diabetes and who were asked for their treatment, 44% (n = 4131) reported to be treated for diabetes (Table 3). For those measured with elevated BP 11% (n = 2599) reported to receive antihypertensive treatment. Of the participants treated for diabetes 46% (n = 3574 out of n = 7705) had normal glucose values, whereas 56% (n = 3282 out of n = 5881) of those treated for hypertension measured normal BP values. Of the participants whose measured BP value classified them as having grade 3 hypertension (n = 1383), only 15.1% received anti‐hypertensive treatment (Table S3).

**TABLE 3:** Participants treated and untreated for hypertension and diabetes

	Diabetes (No. = 12 571)	non‐Diabetes (No. = 72 333)	Total (No. = 84 904)
No.‐Miss	3182	16 361	19 543
Treated	4131 (44.0%)	3574 (6.4%)	7705 (11.8%)
Untreated	5258 (56.0%)	52 398 (93.6%)	57 656 (88.2%)

	Hypertension (No. = 30 345)	Normotension (No. = 61 163)	Total (No. = 91 508)
No.‐Miss	7334	12 257	19 591
Treated	2599 (11.3%)	3282 (6.7%)	5881 (8.2%)
Untreated	20 412 (88.7%)	45 625 (93.3%)	66 037 (91.8%)

No.‐Miss indicates the number of missing values.

### 3.2 Prevalence values of hypertension and diabetes

**FIGURE 2:**
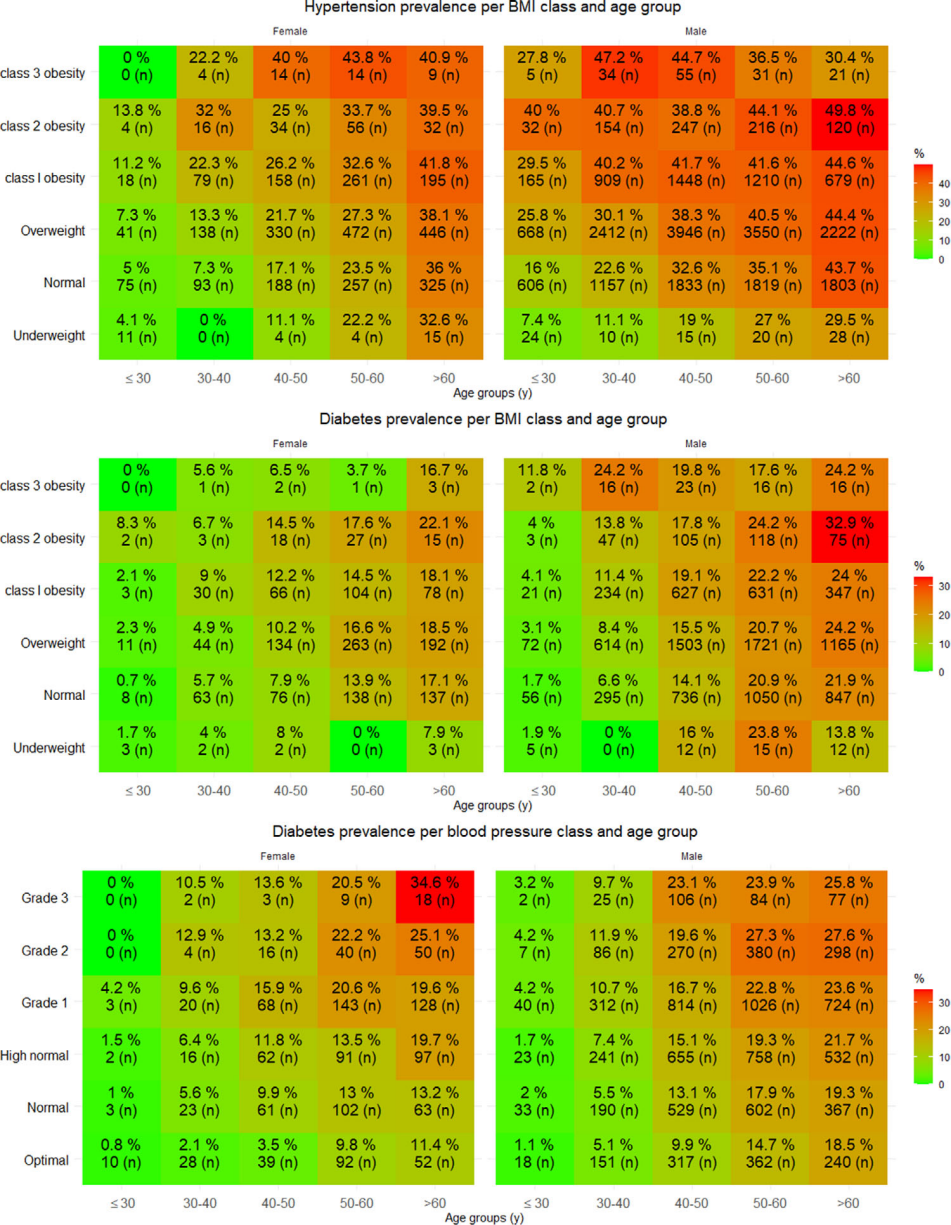
Prevalence of hypertension and diabetes categorized for age groups and BMI class (top and middle picture) and prevalence of diabetes by age‐group and blood pressure class (below), separated by sex. For each category the number of participants affected is presented (n) together with the percentage (%) calculated from the total number of participants per category

Stratification by age‐groups and BMI classes and separated for sex, showed a similar trend in hypertension prevalence for both males and females (Figure 2, top), although males showed higher values overall. Hypertension prevalence increased with higher BMI classes and showed a stepwise increase with each age‐group. High BMI had a major effect on hypertension at younger age in both males and females, in the age groups younger than 40 years old the hypertension prevalence among obese subjects was about twice the prevalence as compared to those with normal BMI. However, this BMI effect seemed to become less with older age. This was confirmed with Pearson correlation analysis showing that systolic BP correlated stronger with BMI in the group younger than 40 years of age than in the group of 40 years and older (*r* = 0.27 vs *r* = 0.06, *p <* .0001, respectively)

Among obese (BMI > 30 kg/m^2^) women aged 60 years and older more than 40% were hypertensive, whereas for obese men a similar hypertension prevalence value of 40% or more was already seen 3 decades earlier in the age‐group of 30‐40 years old.

A similar trend as for hypertension prevalence was seen for diabetes prevalence (Figure 2, middle). Diabetes showed a clear stepwise increase with each higher age‐group. Similar as for hypertension the diabetes prevalence seemed to increase more with higher BMI classes at younger age than at older age. This was confirmed with Pearson correlation analysis that showed that RBG had a higher correlation with BMI for those younger than 40 years of age, as compared to those 40 years and older (*r* = 0.15 vs *r* = 0.03, *p <* .0001, respectively)

Categorization for BP classes and age groups showed a similar stepwise and predictive increasing trend with diabetes prevalence (Figure 2, bottom). Overall, males showed higher diabetes prevalence values than females, but this difference was not as large as was seen for hypertension. When aged older than 50 years and classed hypertension grade 1 or higher both males and females showed a diabetes prevalence of 20% or more.

## 4 DISCUSSION

The present retrospective analysis from data of 100 107 participants screened showed that one‐third had hypertension, 15% had diabetes, and 18% of all participants were obese (65% being overweight). Among those with elevated BP and elevated blood glucose the awareness and treatment rates were low; 44% of the participants measured with elevated blood glucose values received anti‐diabetes treatment and 11% of those with elevated BP received anti‐hypertensive treatment. This suggests that the awareness of diabetes was higher than for hypertension.

Although significant due to the large sample size, correlation values between parameters in the present analysis were generally poor. However, there were some obvious and significant differences in correlation values between younger and older subjects. At young age BMI better correlated with both systolic BP and blood glucose than at older age. Categorization for diabetes and BP showed that diabetes and elevated BP are causally related as the highest diabetes prevalence values were found in the higher BP classes (hypertension grade 1 and 2). Both males and females showed the same pattern, but males showed higher prevalence values for both diabetes and hypertension, overall.

### 4.1 Awareness and treatment of diabetes and hypertension

India is often referred to as a diabetes country. Indians seem more prone to type 2 diabetes than other populations, due to greater degree of insulin resistance and a stronger genetic predisposition to diabetes and also due to obesity, especially central obesity and increased visceral fat due to physical inactivity, and consumption of a high‐calorie/high‐fat and high sugar diets.^14^ This is in line with the findings of the present retrospective analysis in which participants had a high average BMI, with most participants being overweighted and by finding of a moderate correlation between blood glucose level and BMI, in particular at younger age. Altogether, this may have led to the fact that the awareness of diabetes is relatively high among Indians as compared to hypertension. However, the present report also shows that more participants had hypertension than diabetes (30 585 vs 12 420) and that relatively less participants received blood pressure lowering treatment (11% vs 44%, respectively). Our results show the number of Indians affected with hypertension greatly outnumber those with diabetes. Therefore, hypertension, like diabetes, must receive considerable attention to encounter cardiovascular diseases.

The hypertension and diabetes prevalence values of the present screening study are higher but in line with those reported in an earlier performed large population‐based study among 1.3 million Indians. This study showed that diabetes and hypertension prevalence was high with 7.6% and 26.5%, respectively^3^ However, the present screening initiative also provides some additional information to that study, such as the underrepresentation of women and the above‐mentioned results regarding (lack of) anti‐diabetes and anti‐hypertension treatment.

The fact that there were many more males than females screened may have led to overestimation of hypertension and diabetes values. However, it also highlights an important Indian health care problem namely that Indian women are less likely to visit healthcare services as was earlier reported by us^15^ and also other researchers.^16^ Recently, Kapoor and associates who performed an analysis of 2 377 028 outpatient visits in India concluded that there is gender discrimination in access to health care, particularly for female patients in the younger and older age groups.^16^ Awareness campaigns like ours followed by publication of the results might trigger the required systemic societal and governmental action to correct this inequity.

### 4.2 Why screening at the airport?

The idea for taking this screening initiative was triggered by the “May Measurement Month” initiative, a global campaign set up to raise awareness of hypertension and to improve blood pressure screening facilities in those parts of the world where such facilities are particularly poor.^17^ Within this initiative it is highlighted that for screening sites a wide range of locations may be considered. In order to cover a wider spectrum of cardiovascular diseases measurements were expanded for assessing blood glucose and BMI.

The departure hall of an airport appears to be an excellent place for retail business because one can reach a high number of people. This nowadays particularly applies to India. Due to rapid changes in the economy, infrastructure and the start of many low‐cost airlines, Indian air traffic has increased quickly. From 2014 to 2019, the number of passengers handled at airports in India increased from 169 million to 345 million. Interestingly, whereas the number of international flight passengers only increased by 50% during this time, the number of domestic flights increased by more than 300% to 275 million passengers in 2019.^18^ Because more than 80% of all flights in India are domestic, it is not surprising that also the majority of the population screened in the present paper also had an Indian nationality.

The overall aim of this screening set‐up was to increase cardiovascular risk awareness, which basically does not differ much from the aim of creating brand awareness that most retail stores at the airport have. The reason that this has proven its value is because, next to the high number of visitors, the store opening times at airports has a much longer time span than the normal markets. This benefit was recognized for the present screening campaign, with more than 8000 individuals screened before 8 h am and almost 4500 between 9 and 10 h PM.

In addition, airport visitors can easily spend an hour or more in the departure hall with less distraction than usual. During that time, people may be more willing than usual to participate in a screening event that could easily take 20 min of their time. Finally, because people have more time to waste, they may experience less stress than usual. This may have a positive effect on the examination, especially for blood pressure measurement.

### 4.3 Strengths and limitations of the study

The strength of the present retrospective analysis lies in the high number of participants who were screened and the fact that the screening was performed by well‐trained technicians. BP measurement was performed in triplicate with a validated oscillometric device. ^10^ ^,11,19,^ ^20^ One may argue that hypertension cannot be diagnosed by a one‐time triplicate measurement, which is true. Nevertheless, we believe that these figures clearly suggest undertreatment of hypertension for two reasons; First, the figures from the present screening confirm earlier results.^21^ Second, among participants with the highest BP (grade 3), who are unlikely to be normotensive, still only 15% of this group received anti‐hypertensive drugs.

Blood glucose was measured using a blood glucose meter, although the blood glucose monitor may be considered reliable, the manner of testing is not the gold standard for diagnosing diabetes, and therefore the classification “diabetes” based on the measured value might not be appropriate. However, RBG level equal to and above the range of 200 mg/dL has shown to provide good discrimination for follow‐up diagnosis.^22^ The present paper also contains data from hospital visitors, this population clearly deviated from the airport visitors and suggests that these were hospital patients with higher cardiovascular risk, but this was not verified. As the hospital visitors represent only 1.4% of the screening participants, their influence on the overall results is minimal. For statistical analysis we did not adjust for screening location and state of living as this was not within the scope of present paper. However, there are some differences in BMI, blood glucose, and blood pressure values between some states of India as can be seen in Figure S3. There was a large difference in the number of males and females who were screened. A reason for this might be that, next to the reason we have explained earlier, airports are often visited by business people who are, certainly in India, more often male. Finally, although the economic situation is quickly improving, India has a skewed economic distribution. Flight tickets can be relatively cheap, certainly when these are domestic and 80% of the participants screened reported to fly only occasionally, but still most Indians cannot afford plane tickets so that the present data from airport visitors may not be considered as nationally representative. However, the study of Geldsetzer and associates showed that being part of the richest household wealth quintile compared with being in the poorest quintile was associated with only a modestly higher probability of diabetes (2.81 and 3.47 percentage points for rural and urban, respectively) and hypertension (4.15 and 3.01 percentage points for rural and urban, respectively). In addition, education level appeared to have minor influence. This is important information for the interpretation of the results from the present screening as most participants may belong to the richest household quintile of India and have relatively high educational level.^3^

## 5 CONCLUSION

Although, the sample may not be considered as nationally representative, the big data provided important information that may be used for influencing screening policy and the management of cardiovascular diseases in India.^23^ The present screening initiative revealed a high prevalence of important cardiovascular risk factors. In addition, it showed a low level of awareness and treatment of diabetes and for hypertension. However, the treatment level for diabetes was higher than for hypertension (44% vs 11%). These results show that Screening initiatives like these are needed to increase overall awareness of diabetes and particularly of hypertension. Systematic screening programs also help to identify specific patient populations and cope with undertreatment of those at highest cardiovascular risk. For example, the present study showed that the prevalence of diabetes was higher than 15% and 20% and the hypertension prevalence was higher than 25% and 40% in overweight men and women aged 50 years and older, respectively. In addition, both diabetes and hypertension prevalence values were relatively high in young adults with obesity. These findings indicate that preventive health care strategies focusing on these groups would be most efficient to improve cardiovascular health. Finally, the fact that women were underrepresented in the present study suggests that actions are needed to encourage them, in particular, to participate in health care programs.

## CONFLICTS OF INTEREST

WJV is an employee of Microlife corporation, Taipei, Taiwan

NG is an employee of Eris Lifesciences Ltd.

## AUTHOR CONTRIBUTIONS

WJV analyzed data, wrote manuscript, and prepared the figures.

NG, guided the screening project, collected the data, and reviewed the manuscript.

## Supporting information

Supporting material
