# Supplementary material for: Cardiovascular risk factors of airport visitors in India: results from a nation‐wide campaign
Source: J Clin Hypertens (Greenwich). 2021 Dec 13;24(1):74–82. doi: 10.1111/jch.14413 (PMC8783355; doi:10.1111/jch.14413)
Supplement: Supplementary file 1 — Supporting material [file JCH-24-74-s001.docx]

Supplement Airport screening booths

Contents

[Figure S1. Number of subjects screened per state 2](#_Toc85992228)

[Figure S2 Example report of subject’s screening results (SMS) 3](#_Toc85992229)

[Table S1. Subject’s characteristics separated for subjects living in India or elsewhere 4](#_Toc85992230)

[Table S2. Subject’s characteristics from hospital vs. Airport 6](#_Toc85992231)

[Table S3. Hypertension treatment separated for blood pressure classes 7](#_Toc85992232)

[Figure S3. Average Systolic blood pressure, random blood glucose and BMI values per state of India. 8](#_Toc85992233)

# Figure S1. Number of subjects screened per state


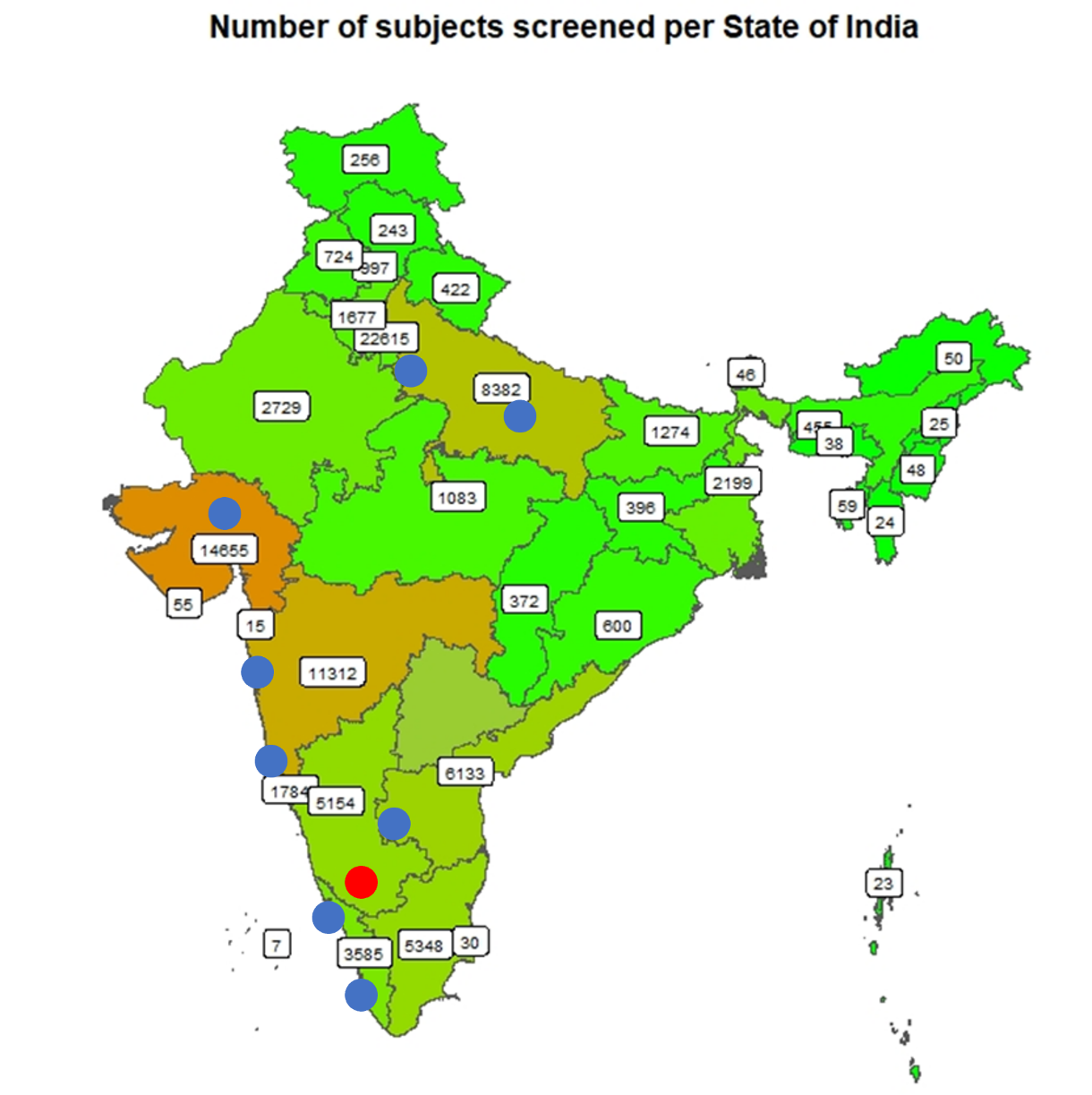


Numbers in the white squares represent the number of subjects screened who were from that state, the blue dots represent the airports where the screening booths were located and the red dot represents the place where the hospital booth was located.

# Figure S2 Example report of subject’s screening results (SMS)


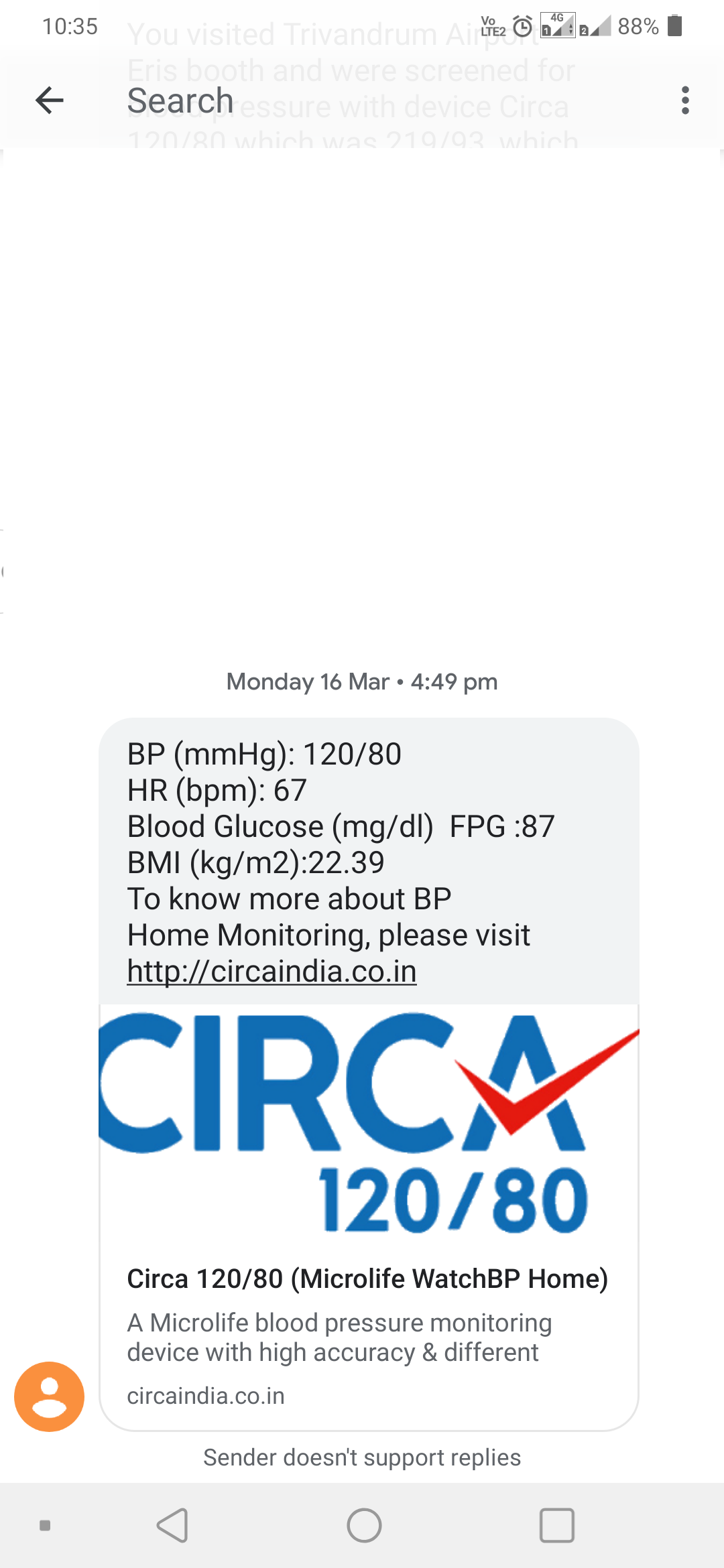


| Table S1. Subject’s characteristics separated for subjects living in India or elsewhere | | | |
| --- | --- | --- | --- |
|  | **Elsewhere (N=817)** | **India (N=100107)** | **p value** |
| **Gender** |  |  | < 0.001 |
| Female | 316 (38.7%) | 17321 (17.3%) |  |
| Male | 501 (61.3%) | 82786 (82.7%) |  |
| **Age** |  |  | < 0.001 |
|  | 49.0 (15.1) | 45.9 (12.9) |  |
| **Systolic BP (mmHg)** |  |  | 0.040 |
|  | 128.3 (17.6) | 129.7 (17.2) |  |
| **Diastolic BP (mmHg)** |  |  | < 0.001 |
|  | 79.5 (11.2) | 82.4 (11.0) |  |
| **Heart Rate (BPM)** |  |  | < 0.001 |
|  | 80.8 (13.3) | 85.4 (13.5) |  |
| **Random Blood Glucose (mg/dL)** |  |  | < 0.001 |
|  | 134.4 (51.3) | 146.5 (64.2) |  |
| **Fasting Plasma Glucose (mg/dL)** |  |  | 0.955 |
|  | 109.8 (25.2) | 110.0 (24.5) |  |
| **BMI** |  |  | < 0.001 |
|  | 26.0 (4.5) | 26.7 (4.0) |  |
| **Blood pressure diagnosis** |  |  | 0.076 |
| Hypertension | 240 (30.2%) | 30345 (33.2%) |  |
| Normotension | 555 (69.8%) | 61164 (66.8%) |  |
| **Diabetes total** |  |  | < 0.001 |
| DM | 61 (9.5%) | 12571 (14.8%) |  |
| non-DM | 580 (90.5%) | 72333 (85.2%) |  |
| **Obese** |  |  | 0.077 |
| Non-Obese | 683 (84.3%) | 77381 (81.9%) |  |
| Obese | 127 (15.7%) | 17074 (18.1%) |  |
| **Heart Rate threshold of 80 BPM** |  |  | < 0.001 |
| <80 | 366 (46.1%) | 31344 (34.4%) |  |
| >80 | 428 (53.9%) | 59804 (65.6%) |  |
| **Treat** |  |  | 0.014 |
| Both | 9 (1.1%) | 2613 (2.6%) |  |
| Diabetes | 35 (4.3%) | 5465 (5.5%) |  |
| Hypertension | 24 (2.9%) | 3408 (3.4%) |  |
| None | 749 (91.7%) | 88621 (88.5%) |  |
| **Hypertension awareness** |  |  | 0.410 |
| No | 774 (94.7%) | 94153 (94.1%) |  |
| Yes | 43 (5.3%) | 5954 (5.9%) |  |
| **Diabetes awareness** |  |  | 0.063 |
| No | 766 (93.8%) | 92087 (92.0%) |  |
| Yes | 51 (6.2%) | 8020 (8.0%) |  |
| **Profession** |  |  | < 0.001 |
| Business person | 157 (23.9%) | 20106 (22.8%) |  |
| Corporate Job | 343 (52.3%) | 51002 (57.9%) |  |
| Education | 15 (2.3%) | 1033 (1.2%) |  |
| Government Service | 16 (2.4%) | 5889 (6.7%) |  |
| House hold | 101 (15.4%) | 8769 (10.0%) |  |
| Student | 24 (3.7%) | 1308 (1.5%) |  |
| **Flying Frequency** |  |  | < 0.001 |
| Multiple times a week | 5 (0.8%) | 1909 (2.2%) |  |
| Occasional | 613 (96.1%) | 68952 (79.7%) |  |
| Once a month | 19 (3.0%) | 12813 (14.8%) |  |
| Once a week | 1 (0.2%) | 2838 (3.3%) |  |

The group “Elsewhere” indicates airport visitors who reported to come from any country outside India. “India” represents visitors from India.

| Table S2. Subject’s characteristics from hospital vs. Airport | | | |
| --- | --- | --- | --- |
|  | **Airports (N=98678)** | **Hospital (N=1428)** | **p value** |
| **Gender** |  |  | < 0.001 |
| Female | 16817 (17.0%) | 504 (35.3%) |  |
| Male | 81861 (83.0%) | 924 (64.7%) |  |
| **Age** |  |  | < 0.001 |
|  | 45.8 (12.8) | 47.8 (13.3) |  |
| **Systolic BP (mmHg)** |  |  | 0.020 |
|  | 129.6 (17.2) | 131.1 (19.5) |  |
| **Diastolic BP (mmHg)** |  |  | < 0.001 |
|  | 82.4 (10.9) | 83.8 (12.3) |  |
| **Heart Rate (BPM)** |  |  | 0.001 |
|  | 85.4 (13.5) | 84.3 (13.3) |  |
| **Random Blood Glucose (mg/dL)** |  |  | 0.133 |
|  | 146.5 (64.1) | 145.1 (67.0) |  |
| **Fasting Plasma Glucose (mg/dL)** |  |  | 0.160 |
|  | 109.8 (24.3) | 119.1 (31.9) |  |
| **BMI** |  |  | < 0.001 |
|  | 26.7 (4.0) | 27.4 (4.8) |  |
| **Blood pressure diagnosis** |  |  | < 0.001 |
| Hypertension | 29790 (33.1%) | 555 (38.9%) |  |
| Normotension | 60291 (66.9%) | 872 (61.1%) |  |
| **Diabetes total** |  |  | 0.963 |
| DM | 12444 (14.8%) | 127 (14.8%) |  |
| non-DM | 71598 (85.2%) | 734 (85.2%) |  |
| **Obese** |  |  | < 0.001 |
| Non-Obese | 76306 (82.0%) | 1074 (75.4%) |  |
| Obese | 16724 (18.0%) | 350 (24.6%) |  |
| **Heart Rate threshold of 80 BPM** |  |  | 0.002 |
| <80 | 30805 (34.3%) | 539 (38.3%) |  |
| >80 | 58933 (65.7%) | 870 (61.7%) |  |
| **Treat** |  |  | < 0.001 |
| Both | 2520 (2.6%) | 93 (6.5%) |  |
| Diabetes | 5315 (5.4%) | 150 (10.5%) |  |
| Hypertension | 3208 (3.3%) | 200 (14.0%) |  |
| None | 87635 (88.8%) | 985 (69.0%) |  |
| **Hypertension awareness** |  |  | < 0.001 |
| No | 92975 (94.2%) | 1177 (82.4%) |  |
| Yes | 5703 (5.8%) | 251 (17.6%) |  |
| **Diabetes awareness** |  |  | < 0.001 |
| No | 90888 (92.1%) | 1198 (83.9%) |  |
| Yes | 7790 (7.9%) | 230 (16.1%) |  |

The group “Hospital” represents all visitors who visited the health care screening booth located at the hospital entrance hall. “Airports” represents the visitors who were screened at the health care screening booths in the airport departure halls.

| Table S3. Hypertension treatment separated for blood pressure classes | | | | | | | |
| --- | --- | --- | --- | --- | --- | --- | --- |
|  | **Optimal (N=20610)** | **Normal (N=20165)** | **High normal (N=20389)** | **Grade 1 (N=21940)** | **Grade 2 (N=6428)** | **Grade 3 (N=1977)** | **Total (N=91509)** |
| **Hyp_Treatment** |  |  |  |  |  |  |  |
| N-Miss | 4343 | 3870 | 4044 | 4998 | 1742 | 594 | 19591 |
| Treated | 896 (5.5%) | 1129 (6.9%) | 1257 (7.7%) | 1732 (10.2%) | 658 (14.0%) | 209 (15.1%) | 5881 (8.2%) |
| Untreated | 15371 (94.5%) | 15166 (93.1%) | 15088 (92.3%) | 15210 (89.8%) | 4028 (86.0%) | 1174 (84.9%) | 66037 (91.8%) |

“Optimal” indicates a systolic and diastolic blood pressure of <120 and <80 mmHg; “Normal”, a systolic and diastolic blood pressure from 120 to 129 mmHg and from 80 to 84 mmHg; “High normal”, from 130 to 139 mmHg and from 85 to 89 mmHg; “Grade 1 hypertension” from 140 to159 mmHg and from 90 to 99 mmHg; “Grade 2 hypertension”, from 160 to 179 mmHg and from 100 to 109 mmHg; “Grade 3 hypertension”, ≥180 mmHg and ≥110 mmHg for systolic and diastolic blood pressure, respectively.

Figure S3. Average Systolic blood pressure, random blood glucose and BMI values per state of India.
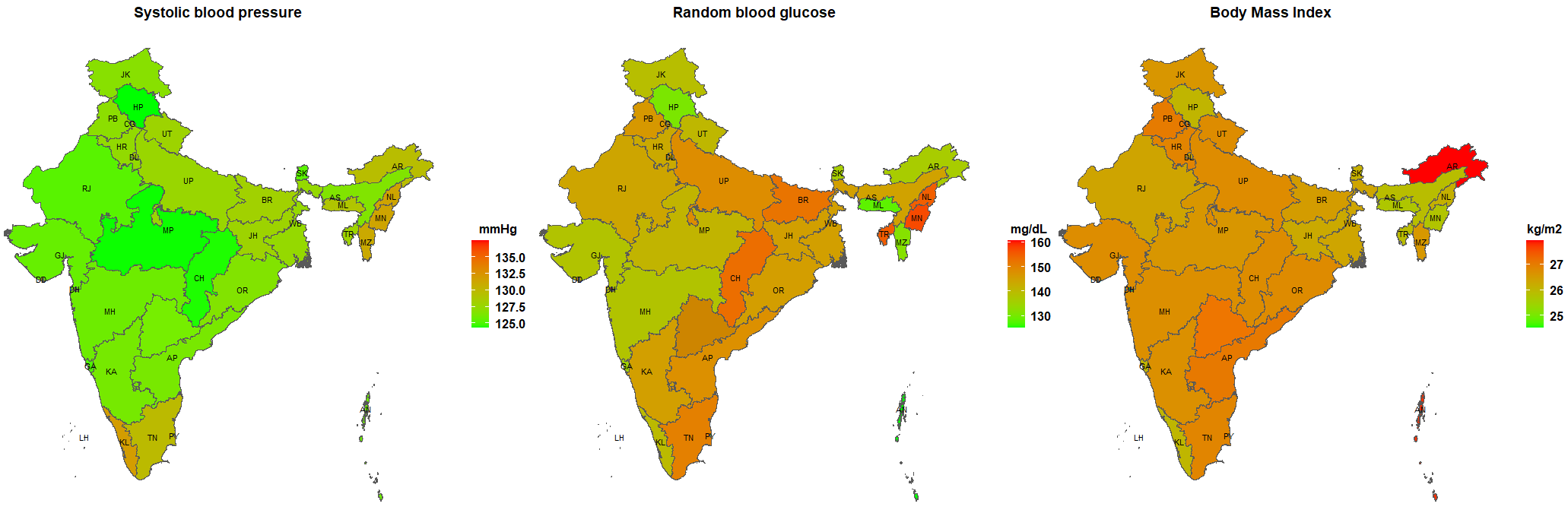


**Figure S3.** Average Systolic blood pressure (left), random blood glucose (middle) and BMI values (right) per state of India. Values were adjusted for gender and age. The legend to the right of each map shows the colours and units that belong to the (average) values.
